# Diagnostic Efficacy and Decision-Making Role of Preoperative Endoscopic Ultrasonography in Early Gastric Cancer

**DOI:** 10.3389/fmed.2021.761295

**Published:** 2021-10-20

**Authors:** Xiao Li, Min Zhu, Yongjun Wang, Yinglin Niu, Ming Ji, Peng Li, Shutian Zhang

**Affiliations:** Department of Gastroenterology, Beijing Friendship Hospital, Capital Medical University, National Clinical Research Center for Digestive Diseases, Beijing Digestive Disease Center, Beijing Key Laboratory for Precancerous Lesion of Digestive Diseases, Beijing, China

**Keywords:** early gastric cancer, endoscopic ultrasonography, invasion depth, diagnostic efficacy, ESD, surgery

## Abstract

**Objectives:** Endoscopic ultrasonography (EUS) is the most commonly used method for T staging of early gastric cancer (EGC). However, the studies pertaining to EUS for staging EGC reported widely varied sensitivities and specificities. This study aimed to estimate the overall diagnostic accuracy of EUS for staging the depth of EGCs and to explore the influential factors.

**Methods:** We retrospectively reviewed data from 208 consecutive patients with EGC, and all patients underwent EUS for estimating tumor invasion depth, followed by either curative surgery or endoscopic submucosal dissection (ESD). The diagnostic accuracy of EUS was evaluated by comparing the final histologic results of resected specimens. The correlation between accuracy of EUS and characteristics of EGC lesion was analyzed.

**Results:** A total of 211 EGC lesions in 208 patients were included. The overall diagnostic accuracy of EUS in assessing the tumor invasion depth of EGC was 55.9%. Multivariate analysis showed that submucosal invasion (OR 2.615; 95% CI 1.203–5.684, *P* = 0.015) was independently associated with misdiagnosis of the depth of EGC and 0-III type lesions (OR 31.495; 95% CI 2.083–476.256, *P* = 0.013) were an independent risk factor for over-diagnosis of invasion depth by EUS. However, EUS was only suitable for lesions within absolute indications for endoscopic resection.

**Conclusions:** The overall accuracy of EUS in diagnosing invasion depth of EGC was relatively low. Thus, EUS is not necessary routinely for determining the therapeutic strategy for EGC.

## Introduction

The long-term outcome and quality of life of patients after endoscopic treatment are not inferior to surgical treatment for early gastric cancer (EGC) (1, 2). Currently, endoscopic treatment, especially endoscopic submucosal dissection (ESD), is widely accepted as a standard treatment for EGC in Japan and Korea, because it is much more minimally invasive. With the indications for endoscopic treatment gradually being expanded, it has become increasingly important in the pre-treatment planning to accurately determine the depth of invasion (3).

Endoscopic ultrasonography (EUS) has been accepted as a useful diagnostic modality for the evaluation of lesions in the gastrointestinal tract and the visceral structures around it. Previous studies demonstrated that EUS was effective in staging of gastric cancer with high accuracy around 90% and sensitivity and specificity of 80–90% (4, 5). However, several other studies drew an opposite conclusion that EUS was not superior to conventional esophagogastroduodenoscopy (EGD) in terms of predicting T staging of EGC (6–9). In addition, previous reports have suggested that the accuracy of EUS was influenced by several factors: endoscopic findings, the location of the lesion, the stage of the gastric cancer, tumor size, EUS type, and study design (9–11).

The objectives of this study were to evaluate the utility of EUS in determining the depth of EGC invasion and to analyze clinicopathological factors influencing the diagnostic accuracy of EUS in predicting the depth of tumor invasion.

## Methods

### Participants

Three hundred and twelve lesions underwent curative surgery or ESD from January 2015 to December 2017 in our center, with 289 of them diagnosed with EGCs pathologically. Two hundred and eight patients (211 lesions) underwent pretreatment EUS and we reviewed the endoscopic images of these patients. Of these 211 lesions investigated in the present study, 131 underwent ESD successfully and 20 underwent surgery after ESD procedure because of ESD failure in five patients and uncompleted resection in 15 patients, and the remaining 60 patients underwent surgery primarily. This study was conducted in accordance with the Helsinki Declaration and approved by the Beijing Friendship Hospital Ethics Committee.

### Equipment and Examination Procedures

All lesions were observed by conventional endoscopy (video endoscope Q260 or H260, Olympus Medical Systems, Tokyo, Japan) followed by EUS. Two kinds of EUS devices were used: the ultrasound probe (US-probe; UM-3R, 20 MHz, Olympus, Tokyo, Japan) was selected for use with smaller or flat lesions, and the ultrasound endoscope (US-endoscope; EG-530UT, EG-530UR, FUJI, Japan) was selected for use with larger or depressed lesions. EUS examinations were performed by three endoscopists (PL, YW, and YN) with more than 5 years of EUS experience.

### Definitions

#### Endoscopic Findings

Based on the location of the lesion under endoscopy, gastric lesions were divided into four groups: the cardia/fundus, the body of the stomach, the angle of the stomach, and the antrum of the stomach. The macroscopic features of the lesions were classified as protruded type (0-I), superficial type (0-II, including IIa, IIa + IIc, IIb, IIc, and IIc + IIa), and excavated type (0-III) based on Paris endoscopic classification of superficial neoplastic lesions (12). Endoscopically, the presence of ulcer was determined as active ulceration with thick whitish necrotic exudate in the active stage or as mucosal convergence in the healing stage. Roughness surface was defined when there were nodules on the tumor's surface.

#### Histopathology Evaluation

Histopathological evaluation was performed by sections of 2 millimeters thickness for endoscopic resection, and 4 mm thickness for surgical resection stained with hematoxylin and eosin. According to post-operative histological assessment, each lesion was classified as either differentiated type [including well-differentiated tubular adenocarcinoma (tub1), moderately differentiated tubular adenocarcinoma (tub2) and papillary adenocarcinoma (pap)], or undifferentiated type [including poorly differentiated adenocarcinoma (por), signet ring cell carcinoma (sig), and mucinous cell carcinoma (muc)]. The lesions were divided into the histological mucosal (M) group and the submucosal (SM) group. Based on the Japanese Gastric Cancer Treatment Guideline, lesions were categorized into three groups according to tumor-related factors: within absolute indication, expanded criteria, and beyond expanded criteria. The absolute indications were mucosal cancer, differentiated-type adenocarcinoma, ulcer (–), and ≤2 centimeters (cm) in diameter. The expanded criteria were (1) mucosal cancer, differentiated-type adenocarcinoma, ulcer (–), and any tumor size; (2) mucosal cancer, differentiated adenocarcinoma, ulcer (+), and ≤3 cm in size; and (3) mucosal cancer, ulcer (–), undifferentiated carcinomas, and <2 cm in diameter (3).

#### Identification of Cancer Invasion Depth by EUS

During EUS, the gastric wall was assessed based on the standard five-layer sonographic structure. Cancer depth was evaluated as M if the hypoechoic mass disrupted the sonographic layers 1–2, as SM if it disrupted layers 1–3, as muscularis propria (MP) if layers 1–4 were disrupted, and as subserosa and serosa (SS) if layers 1–5 were discontinued.

### Statistical Analysis

Continuous variables were expressed as means ± standard deviation (SD) or median (range) depending on whether they fit the normal distribution or not, and categorical variables were presented as proportions. The diagnostic accuracy, sensitivity, specificity, positive predictive value (PPV), and negative predictive value (NPV) were calculated using standard definitions.

The endoscopic and histopathological findings were analyzed to determine if they influenced the EUS diagnosis of the depth of cancer invasion. SPSS package version 21.0 (SPSS, Chicago, IL, USA) was used for statistical analysis. A Chi-square test was used for the univariate analyses, and logistic regression was used for multivariate analyses. Two-sided *P* < 0.05 was considered statistically significant.

## Results

### Baseline Characteristics of Enrolled Patients

A total of 208 patients (211 lesions) were finally analyzed. The median age of the patients was 63 years old (Rang, 32–84), and the proportion of male was 70.7%. The mean body mass index (BMI) was 24.0 Kg/m^2^ (mean ± SD, 24.0 ± 3.2). The median tumor diameter was 2.0 cm (Range, 0.4–10.0) and ulcer/scar was accompanied in 27.0% lesions. In the final pathological diagnosis, the invasion depth was M for 149 lesions (70.6%) and SM for 62 lesions (29.4%), and differentiated histology was diagnosed in 76.3% and undifferentiated histology in 23.7% (Table 1).

**Table 1:** The clinicopathological characteristics of subjects.

**Patients' characteristics (*n* = 208)**	
Male/Female, *n* (%)	147 (70.7)/61 (29.3)
Age, median (range) (years)	63 (32–84)
BMI, mean ± SD (Kg/m^2^)	24.0 ± 3.2
**Lesion characteristics (*****n*** **= 211)**	***N*** **(%)**
**Location**	
Cardia/fundus	36 (17.1)
Body	45 (21.3)
Angle	32 (15.2)
Antrum	98 (46.4)
Tumor diameter^*^	
Median (range) cm	2.0 (0.4–10.0)
≤ 2.0 cm	98 (46.6)
>2.0 cm	105 (49.8)
**Macroscopic type**	
0–I	9 (4.3)
0–II	158 (74.9)
0–III	44 (20.9)
**Ulcer/Scar**	
Positive	57 (27.0)
Negative	154 (73.0)
**Surface roughness**	
Positive	200 (94.8)
Negative	11 (5.2)
**White fur**	
Negative	127 (60.2)
Mediate	46 (21.8)
Much	38 (18.0)
**Pathologic depth**	
M	149 (70.6)
SM	62 (29.4)
**Histology**	
Differentiated	161 (76.3)
tub1/tub2/pap	118 (55.9)/34 (11.1)/9 (4.3)
Undifferentiated	50 (23.7)
por/sig/muc	14 (6.6)/32 (15.2)/4 (1.9)
**EUS type**	
US-probe	160 (75.8)
US-endoscope	51 (24.2)
**Resection method**	
ESD	131 (62.1)
Surgery after ESD	20 (9.5)
Surgery	60 (28.4)
Indications^**^	
Absolute indication	80 (37.9)
Expanded indication	52 (24.6)
Out of indication	74 (35.1)

* *Missing data in 8 patients (3.8%)*.

** *Missing data in 5 patients (2.4%)*.

*BMI, body mass index; SD, standard deviation; ESD, endoscopic submucosal dissection; EUS, endoscopic ultrasonography; cm, centimeter; M, mucosal; SM, submucosal; tub1, well-differentiated tubular adenocarcinoma; tub2, moderately differentiated tubular adenocarcinoma; pap, papillary adenocarcinoma; por, poorly differentiated adenocarcinoma; sig, signet ring cell carcinoma; muc, mucinous cell carcinoma*.

### Diagnostic Accuracy of EUS in Assessing the Tumor Invasion Depth

Within the mucosal cancer group, 99 lesions (66.4%) were accurately diagnosed as EUS-M. Out of these 99 lesions, 30 were diagnosed as EUS-SM, 10 as EUS-MM, and the remaining lesions were diagnosed as EUS-SS (*n* = 10). Among the submucosal cancer group, only 19 lesions (30.6%) were accurately diagnosed by EUS. However, 22 lesions (35.4%) were under-estimated as M cancer and 21 lesions (33.9%) were over-estimated either as MP (*n* = 8) or SS (*n* = 13). The overall diagnostic accuracy of EUS in assessing the tumor invasion depth of EGC was 55.9%. We tended to over-stage by EUS more frequently than under-stage (33.6 vs. 10.4%). The sensitivity, specificity, PPV, and NPV for mucosal cancer were 66.4, 64.5, 81.8, and 44.4%, respectively (Table 2).

**Table 2:** Accuracy rates for T staging by endoscopic ultrasonography (*n* = 211).

**T stage by EUS**	**Pathologic stage (** ***n*** **, %)**	
	**M**	**SM**
M	99 (81.8)	22 (18.2)
SM	30 (61.2)	19 (38.8)
MP	10 (55.6)	8 (44.4)
SS	10 (43.5)	13 (56.5)

*EUS, endoscopic ultrasonography; M, mucosa; SM, submucosa; MP, muscularis propria; SS, subserosa and serosa*.

### Influential Factors for Diagnosis Accuracy of EUS

Depending on the endoscopic and histopathological findings, the accuracy of EUS varied widely. The univariate analysis showed that the accuracy was significantly lower for the lesions located at angle and body of the stomach, ulcer/scar (+), excavated type, lesions with white fur on surface, >2.0 cm in diameter, and submucosal invasion, as well as the undifferentiated types of lesions (Table 3). Multivariate analysis of these seven factors showed that submucosal invasion (OR 2.615; 95% CI 1.203–5.684, *P* = 0.015) was independently associated with misdiagnosis of the depth of EGC by EUS (Table 4).

**Table 3:** The accuracy, over-staging, and under-staging rates of EUS for diagnosing EGC invasion depth and univariate analysis of risk factors affecting the diagnostic performance of EUS.

**Lesion characteristics**	**Accuracy**	***P*-value**	**Over-staging**	***P*-value**	**Under-staging**	***P*-value**
Location		0.001		<0.001		0.653
Cardia/fundus	69.4%		19.4%		11.1%	
Body	40.0%		53.3%		6.7%	
Angle	34.4%		50.0%		15.6%	
Antrum	65.3%		24.5%		10.2%	
Tumor diameter^*^		0.025		0.087		0.324
≤ 2.0 cm	63.3%		28.6%		8.2%	
>2.0 cm	47.6%		40.0%		12.4%	
Macroscopic type		<0.001		<0.001		0.137
0–I	77.8%		11.1%		11.1%	
0–II	63.9%		23.4%		12.7%	
0–III	22.7%		75.0%		2.3%	
Ulcer/Scar		<0.001		<0.001		0.632
Positive	31.6%		59.6%		8.8%	
Negative	64.9%		24.0%		11.0%	
Surface roughness		0.597		0.646		0.882
Positive	55.5%		34.0%		9.1%	
Negative	63.6%		27.3%		10.5%	
White fur		0.002		<0.001		0.109
Negative	65.4%		24.4%		10.2%	
Mediate	37.0%		58.7%		4.3%	
Much	47.4%		34.2%		18.4%	
Pathologic depth		<0.001		0.965		<0.001
M	66.4%		33.6%		0	
SM	30.6%		33.9%		35.5%	
Histology		<0.001		<0.001		0.241
Differentiated	64.0%		24.2%		11.8%	
Undifferentiated	30.0%		64.0%		6.0%	
EUS type		0.632		0.775		0.223
US-probe	55.0%		33.1%		11.9%	
US-endoscope	58.8%		35.5%		5.9%	

* *Missing data in 8 patients (3.8%)*.

*EUS, endoscopic ultrasonography; EGC, early gastric cancer; M, mucosa; SM, submucosa*.

**Table 4:** Multivariate analysis of factors associated with misdiagnosis of EUS in estimating the depth of EGC invasion.

	**OR**	**95% CI**	***P*-value**
**Location**			
Cardia/fundus	1	–	–
Body	1.162	0.305, 4.433	0.826
Angle	2.079	0.661, 6.542	0.211
Antrum	1.004	0.422, 2.391	0.992
Tumor diameter^*^			
≤ 2.0 cm	1	–	–
>2.0 cm	1.415	0.748, 2.675	0.286
**Macroscopic type**			
0–I	1	–	–
0–II	1.512	0.274, 8.334	0.635
0–III	7.210	0.742, 70.061	0.089
**Ulcer/Scar**			
Negative	1	–	–
Positive	0.841	0.228, 3.099	0.795
**White fur**			
Negative	1	–	–
Mediate	0.890	0.297, 2.669	0.836
Much	1.552	0.650, 3.703	0.322
**Pathologic depth**			
M	1	–	–
SM	2.615	1.203, 5.684	0.015
**Histology**			
Differentiated	1	–	–
Undifferentiated	1.331	0.386, 4.591	0.651

* *Missing data in 8 patients (3.8%)*.

*EUS, endoscopic ultrasonography; EGC, early gastric cancer; OR, odds ratio; 95%CI, 95% confidence interval; M, mucosa; SM, submucosa*.

In addition, we tended to over-stage more frequently in diagnosing the lesions with the features that were located at angle and body of the stomach, excavated type, ulcer/scar (+), white fur (+), and undifferentiated types. However, type-III was the only independently influential factor for over-staging of EUS in diagnosing EGC invasion depth in multivariate analysis (Table 5).

**Table 5:** Multivariate analysis of risk factors for over-staging of EUS for diagnosing invasion depth of EGC.

**Variate**	**OR**	**95% CI**	***P*-value**
**Location**			
Cardia/fundus	1		
Body	1.143	0.292,4.472	0.848
Angle	1.891	0.567,6.299	0.300
Antrum	0.979	0.369,2.598	0.966
**Macroscopic type**			
0–I	1		
0–II	2.541	0.299,21.573	0.393
0–III	31.495	2.083,476.256	0.013
**Ulcer/Scar**			
Positive	0.382	0.075,1.939	0.246
Negative	1		
**White fur**			
Negative	1		
Mediate	1.305	0.434,3.922	0.636
Much	1.026	0.409,2.574	0.956
**Histology**			
Differentiated	1		
Undifferentiated	1.988	0.623,6.338	0.246

*EUS, endoscopic ultrasonography; EGC, early gastric cancer; OR, odds ratio; 95%CI, 95% confidence interval; M, mucosa; SM, submucosa*.

The accuracy of EUS was analyzed according to the indications for endoscopic resection. The accuracy of EUS for the lesions within absolute indications and expended indications were 78.8 and 57.7%, respectively. For the lesions beyond the indications for endoscopic resection, the accuracy of EUS was 28.4%. There were significant differences in the accuracy among each indication. The frequency of over-staging tended to increase in lesions that escaped the absolute indications (*p* < 0.01). Under-staging was quite low for lesions within absolute indications and within expended indications (0/80 and 0/52, respectively) (Table 6).

**Table 6:** Accuracy of EUS for predicting cancer invasion depth according to the indications for endoscopic resection.

Indications^**^	***n* (%)**	Accuracy^*^	Over-staging^*^	Under-staging^*^
Absolute indications	80 (37.9)	63 (78.8)	17 (21.3)	0
Expanded indications	52 (24.6)	30 (57.7)	22 (42.3)	0
Out of indications	74 (35.1)	21 (28.4)	31 (41.9)	22 (29.7)

* *P < 0.05*.

** *Missing data in 5 patients (2.4%)*.

## Discussion

Although computed tomography (CT) scan has been conventionally used for TNM staging of gastric cancer, CT has shown low diagnostic accuracy in T staging (13). Moreover, CT cannot discriminate the depth of tumor invasion between mucosa and submucosa (14), which is indispensable for the decision-making of treatment modality for EGC. At the time of its introduction in the early 80s, EUS was indicated for diagnostic purposes. Currently, EUS, which has the ability to visualize the tomographic structure of gastric walls, is considered to be the most reliable diagnostic modality used to predict the depth of gastric cancer. Though previous studies have proven the clinical efficacy of EUS in T staging of gastric cancer, the results have been inconsistent, especially in EGC, ranging from 45 to 92% (7, 8, 15, 16). In the present study, we retrospectively investigated the diagnostic ability of EUS in EGC; the overall accuracy of the EUS in assessing the tumor invasion depth was 55.9% and the accuracy rates of EUS for mucosal cancer and submucosal cancer were 66.3 and 30.6%, respectively. The results were consistent with some of the previous studies (8, 16).

It has been reported that the accuracy of EUS tended to decline for the lesions with ulcer (9, 10, 17–19), location in the upper third (17), and those of large tumor size (9, 10, 17, 18). In this study these factors also led to the misdiagnosis of EGC invasion. In previous studies, the stomach was anatomically divided into three portions, namely the upper, middle, and lower thirds of the stomach based on the classification system of the Japanese Gastric Cancer Association (20). However, it is difficult to accurately identify the location of gastric lesions endoscopically according to this classification. As a result, stomach lesions in the current study were divided into four groups, namely the cardia/fundus, the body of the stomach, the angle of the stomach, and the antrum of the stomach, which may be more convenient in the performance of endoscopy. The results showed that the accuracy significantly declined in the lesions located in the body and angle of the stomach as compared with the other locations. In practice, lesions in the body and angle of the stomach, where adequate filling with water is not possible, are difficult to be assessed by EUS.

Ulcer shape was an important factor that affected EUS accuracy (9, 10, 19). In the present study, the accuracy of EUS in evaluating the invasion depth of EGCs with ulcer or scar was extraordinarily low: only 31.6% exactly. More than half of the lesions with ulcer or scar (59.6%) were over-estimated. Compared with mucosal cancers which were correctly diagnosed, those over-estimated mucosal cancers were much more likely to be macroscopically ulcerated (30.0 vs. 7.1%, *p* < 0.01). When ulcers or fibrotic lesions are present, the depth of invasion is most likely misinterpreted as tumor invasion under EUS. For another reason, we conventionally believe that ulcer (+) is one of the indications of submucosal invasion. As a result, when we had difficulty distinguishing lesions by EUS, we tended to diagnose a lesion with ulcer as submucosal invasion because of the interference of conventional EGD findings. For the same reasons, 75% 0-III type lesions were over-staged by EUS in the current study.

White fur on lesions' surface was found to be a factor influencing the diagnosing ability of EUS, which had not been reported before. However, most (92.7%) (data not shown) lesions without white fur on surface were ulcer/scar negative. This result may explain why this characteristic was not an independent risk factor for misdiagnosis of EGC invasion depth in multivariate analysis.

Concerning the pathohistological findings, invasion depth and differentiation degree were related with accuracy in the univariate analysis in the present study. EUS had a higher accuracy in diagnosing mucosal cancers and differentiated lesions than submucosal cancers and undifferentiated cancers, respectively. What's more, submucosal invasion was the only independent risk factor for misdiagnosis of EGC invasion depth by EUS, which statistically indicates that EUS is less useful for confirming the diagnosis of submucosal invasion.

The frequency of the US-probe is higher than that of the US-endoscope, so the US-probe is much more suitable for the determination of the depth of EGC (8, 18). In the current study, the US-probe was used for smaller lesions or lesions with shallower depressions that were easy to diagnose as mucosal cancer, whereas the US-endoscope was used for lesions with a deep ulceration that were difficult to distinguish between a benign fibrosis and a cancerous invasion. However, the accuracy was not significantly different between US-probe and US endoscope group in the current study.

In the case of underestimation, additional surgery after ESD is indispensable because of the high risk of uncompleted resection or un-curative resection. However, unnecessary surgical resection may be an overtreatment in the case of overestimation. Additionally, a previous study illustrated that conventional endoscopy alone has a sufficient diagnostic accuracy in predicting tumor depth in EGC, with an overall diagnostic accuracy of 73.7%, which was significantly higher than that of EUS (8). Based on the fact that the overall accuracy of EUS in the diagnosis of invasion depth of EGC is relatively low, we think EUS could not well-improve the selection of treatment method in EGCs overall. Therefore, EUS may not be necessary routinely for treatment of EGCs.

There were some limitations in the present study. First, this was a non-randomized retrospective designed study at a single tertiary hospital, leading to the possibility of selection bias; therefore, our results may not be generalizable. Second, EGD findings might affect EUS results because the endoscopists were not blinded to the EGD findings in the current study.

In conclusion, the overall accuracy of EUS in diagnosing invasion depth of EGC was relatively low. Submucosal cancers were independently associated with misdiagnosis of the depth of EGC by EUS and 0-III type lesions were an independent risk factor for over-diagnosis of invasion depth by EUS. Thus, EUS is not necessary routinely for determining the therapeutic strategy for EGC.

## Data Availability Statement

The original contributions presented in the study are included in the article/supplementary material, further inquiries can be directed to the corresponding author/s.

## Ethics Statement

The studies involving human participants were reviewed and approved by Beijing Friendship Hospital Ethics Committee. The patients/participants provided their written informed consent to participate in this study.

## Author Contributions

PL and SZ conceived the study. YW, YN, MJ, PL, and SZ designed the study. XL, MZ, and YW contributed to data collection and data analysis. XL and MZ wrote the manuscript. All authors have read and approved the final manuscript.

## Funding

This study was supported by grants from the Digestive Medical Coordinated Development Center of Beijing Municipal Administration of Hospitals (XXZ01 and XXZ02), National Key Research and Development Program of China (2017YFC0113600), National Natural Science Foundation of China (81970490 and 81570507), and Beijing-Tianjin-Hebei Region Collaborative Innovation Promoting Project Founded by Beijing Science and Technology Commission (Z171100004517009).

## Conflict of Interest

The authors declare that the research was conducted in the absence of any commercial or financial relationships that could be construed as a potential conflict of interest.

## Publisher's Note

All claims expressed in this article are solely those of the authors and do not necessarily represent those of their affiliated organizations, or those of the publisher, the editors and the reviewers. Any product that may be evaluated in this article, or claim that may be made by its manufacturer, is not guaranteed or endorsed by the publisher.
